# Penetrating Keratoplasty for Keratoconus – Excimer Versus Femtosecond Laser Trephination

**DOI:** 10.2174/1874364101711010225

**Published:** 2017-07-31

**Authors:** Berthold Seitz, Achim Langenbucher, Tobias Hager, Edgar Janunts, Moatasem El-Husseiny, Nora Szentmáry

**Affiliations:** 1 Department of Ophthalmology, Saarland University Medical Center UKS, Homburg/Saar, Germany; 2 Institute of Experimental Ophthalmology, University of Saarland, Homburg/Saar, Germany

**Keywords:** Keratoconus, Penetrating keratoplasty, PKP, Excimer laser, Femtosecond laser, Trephination, Advantages, Pitfalls

## Abstract

**Background:**

In case of keratoconus, rigid gas-permeable contact lenses as the correction method of first choice allow for a good visual acuity for quite some time. In a severe stage of the disease with major cone-shaped protrusion of the cornea, even specially designed keratoconus contact lenses are no more tolerated. In case of existing contraindications for intrastromal ring segments, corneal transplantation typically has a very good prognosis.

**Methods:**

In case of advanced keratoconus – especially after corneal hydrops due to rupture of Descemet’s membrane – penetrating keratoplasty (PKP) still is the surgical method of first choice. Noncontact excimer laser trephination seems to be especially beneficial for eyes with iatrogenic keratectasia after LASIK and those with repeat grafts in case of “keratoconus recurrences” due to small grafts with thin host cornea. For donor trephination from the epithelial side, an artificial chamber is used. Wound closure is achieved with a double running cross-stitch suture according to Hoffmann. Graft size is adapted individually depending on corneal size („as large as possible – as small as necessary“). Limbal centration will be preferred intraoperatively due to optical displacement of the pupil. During the last 10 years femtosecond laser trephination has been introduced from the USA as a potentially advantageous approach.

**Results:**

Prospective clinical studies have shown that the technique of non-contact excimer laser PKP improves donor and recipient centration, reduces “vertical tilt” and “horizontal torsion” of the graft in the recipient bed, thus resulting in significantly less “all-sutures-out” keratometric astigmatism (2.8 vs. 5.7 D), higher regularity of the topography (SRI 0.80 vs. 0.98) and better visual acuity (0.80 vs. 0.63) in contrast to the motor trephine. The stage of the disease does not influence functional outcome after excimer laser PKP. Refractive outcomes of femtosecond laser keratoplasty, however, resemble that of the motor trephine.

**Conclusions:**

In contrast to the undisputed clinical advantages of excimer laser keratoplasty with orientation teeth/notches in keratoconus, the major disadvantage of femtosecond laser application is still the necessity of suction and applanation of the cone during trephination with intraoperative pitfalls and high postoperative astigmatism.

## 1 INTRODUCTION

**Fig. (1):**
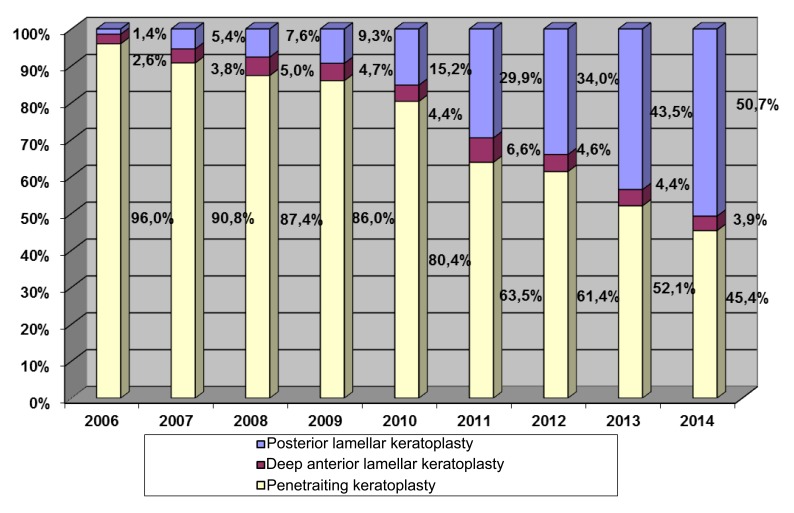
According to the German Keratoplasty Register, which has been maintained for nearly 15 years by the DOG-Sektion Kornea, 50.7% of all cornea transplantations were posterior lamellar, only 3.9% anterior lamellar and still 45.4% were penetrating keratoplasties in 2014.

In the case of keratoconus, rigid gas-permeable lenses have been the first-line correction method for good vision results. However, even special keratoconus lenses can no longer be tolerated after a certain level of cone-shaped deformation of the cornea [1]. If there are also contraindications for intrastromal ring segments [2], corneal transplants are indicated at this stage of the disease, and typically have a very good prognosis [3]. Corneal grafting is the oldest, most common and most successful transplantation in humans [4]. About 40,000 keratoplasties are performed per year in the US. Accordingly, the German Keratoplasty Register that has been maintained for nearly 15 years by the DOG-Sektion Kornea, shows the number of annual keratoplasties in Germany at about 6,000 per year since 2014. 424 were performed in Homburg/Saar in 2016. In the year 2014, 50.7% of all corneal transplants were of the posterior lamellar type, with only 3.9% being anterior lamellar grafts (DALK) and 45.4% still being carried out as penetrating keratoplasties (PKP) (Fig. **1**).

**Fig. (2):**
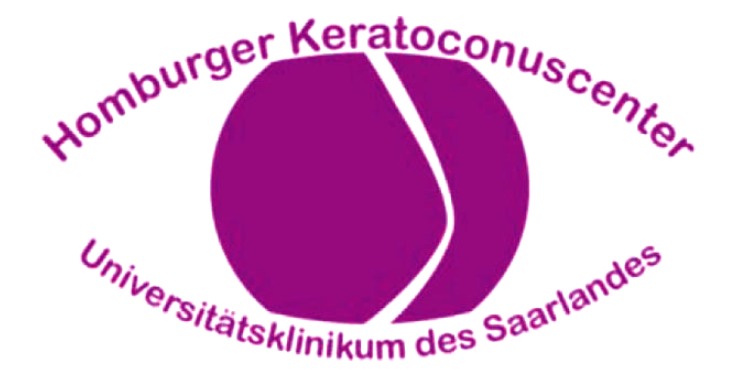
LOGO of the Homburg Keratoconus Centre **HKC**.

 Still, a lack of donated tissues with long waiting lists for keratoplasty presents an unsolved problem, above all for those young patients who are in the midst of their professional years between the ages of 20 and 40, who deal with psychological and economic problems of keratoconus. The indication for keratoplasty with keratoconus has fortunately become more rare after the introduction of Riboflavin UV A Crosslinking [5]. The Homburg Keratoconus Centre, HKC, was founded in 2009 [6]. Its primary goal is to perform standard examinations on all patients in the early stages of keratoconus [7-10], to research the causes and the course of keratoconus, as well as to offer patients stage-related therapy [1-3, 11]. By February 2017, 850 patients have been included in the HKC (Fig. **2**).

### 1.1 Basic Considerations in Penetrating Keratoplasty (PKP)

Today, a clear graft after normal risk keratoplasty in keratoconus with high and/or irregular astigmatism can no longer be considered “successful” [12-14]. There are various pre-, intra- and post-operative causes of high and/or irregular astigmatism in keratoplasty. The three most significant intra-operative determinants of astigmatism after keratoplasty are [12, 15]:

1. **Decentration**, especially with host trephination [16-18].
2. **“Horizontal torsion”,** *i.e.* the second cardinal suture is not placed exactly 180° opposite to the first ccardinal suture [17].
3. “**Vertical tilt**” *i.e.* non-congruent cut edges are compensated by suture tension in order to achieve a water-tight wound closure [17, 19].

### 1.2 Recognising and Treating Underlying Systemic Diseases and Eyelid Abnormalities

As a matter of principle, systemic underlying diseases such as rosacea or neurodermitis - in which problems with the surface of the eye are very common - must be identified and consistently treated before keratoplasty. In cases of very severe neurodermatitis Fig. (**3**), consideration should be given as to whether cyclosporin A orally can be administered at a dosage of 150 mg twice a day for 4 weeks before keratoplasty [12, 13]. Classical eyelid margin hygiene and a dermatological consultation are obligatory.

### 1.3 Individually Optimal Graft Size (“as Large as Possible, as Small as Necessary”)

As a matter of principle, an individually optimised graft size should be selected for each keratoplasty. The graft size is determined pre-operatively for each individual, e.g. using a slit lamp with a measuring device. Each graft should be as large as possible (for optical reasons) and as small as necessary (for immunological reasons). Mostly, transplants ranging from 8.0 to 8.5 mm are ideal for keratoconus [12, 14, 20].

### 1.4 No Keratoplasty in the Acute Stage of Keratoconus (“Corneal Hydrops”)

**Fig. (4):**
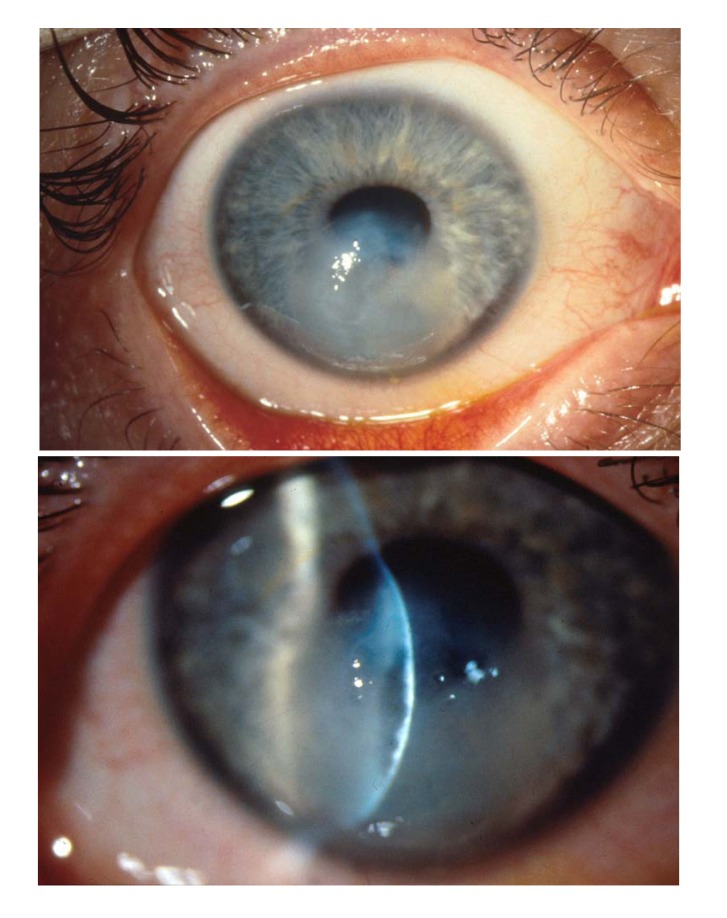
Acute keratoconus (“corneal hydrops”) with a rupture of the Descemet’s membrane. No keratoplasty in the acute stage!.

Immediate penetrating keratoplasty in the acute stage of keratoconus resulting from rupture of Descemet’s membrane (so-called “corneal hydrops”) (Fig. **4**) should be avoided, because suture anchoring in gelatinous tissue is insufficient, and one can regularly count on post-operative thread loosening and corresponding adverse consequences, such as infectious infiltration, neovascularisation and immune responses. The prevalent fear of the doctor and patient of penetration is largely unjustified in corneal hydrops! Nourishing and decongestant drugs are topically applied, and keratoplasty is carried out after 3 to 6 months with good success, after the previously oedematous areas of the cornea have become sufficiently scarred and solid [13, 21]. A Deep Anterior Lamellar Keratoplasty (DALK) using the Big Bubble Technique does not make sense here, because the air would readily enter the anterior chamber via the original Descemet defect.

### 1.5 Intra-Operative Pearls of PKP in Keratoconus

Intubation anaesthesia has safety advantages over local anaesthesia, especially in young keratoconus patients. The arterial blood pressure should be kept as low as possible when the eye is open (“controlled arterial hypotension” with maximum relaxation - do not use mivacurium as a non-depolarising muscle relaxant) [22]). In children, consideration should be given to the pre-operative intravenous administration of diamox and mannitol [23]. The upper body is positioned at about 30° during surgery. In every case the anaesthetist should have been trained in the specific aspects of penetrating keratoplasty before a large opening is made in the globe.

Typically, the pupil is constricted with pilocarpine in order to protect the lens of the phakic eye.

Horizontal positioning of the head and limbal plane is an indispensable precondition for the avoidance of decentration, “vertical tilt” and “horizontal torsion” [17].

Paracentesis at the limbus is recommended before trephination.

In cases of doubt, limbal centration should be preferred over pupil centration, because the optical displacement of the pupil must be taken into consideration [24]. In no case is the goal to “cut out the cone” thereby decentering the excision in the lower temporal direction.

Donor and recipient trephination should be performed from the epithelial side with the same system. This is the prerequisite for congruent cut surfaces and angles in the donor and recipient. An artificial anterior chamber is typically used today for donor trephination [13].

Orientation structures in donor and recipient facilitate the correct placement of the first four or eight cardinal sutures and thereby contribute towards preventing “horizontal torsion”. The second cardinal suture is absolutely crucial for a correct graft fit [13].

It is clearly not advised to under-size the graft in comparison to the recipient bed to compensate myopia, because with that, no tension-free adjustment of the transplant is possible [25], resulting in relative cornea plana and irregular astigmatism.

**Fig. (5):**
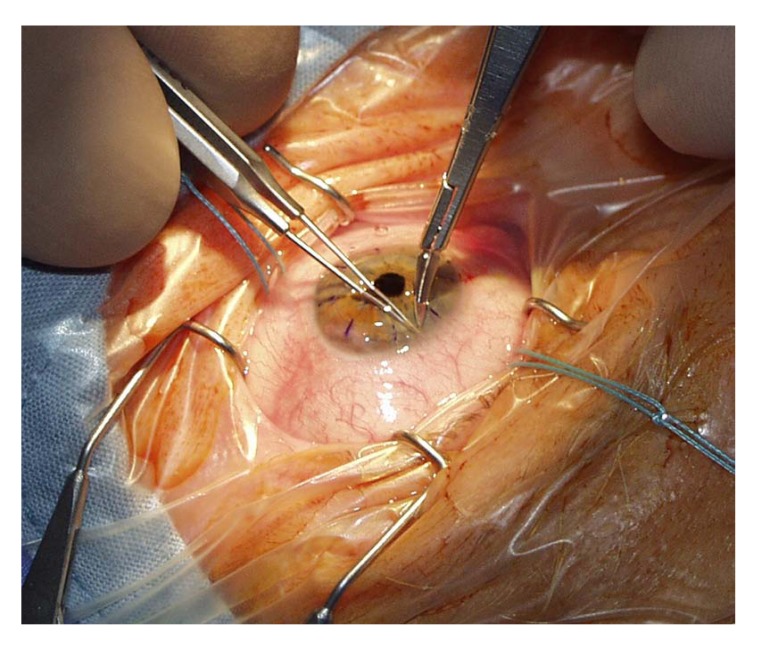
Open-sky iridotomy at 12 o’clock peripherally (no iridectomy needed! CAUTION: Rip of the iris root with substantial bleeding!).

**Fig. (6):**
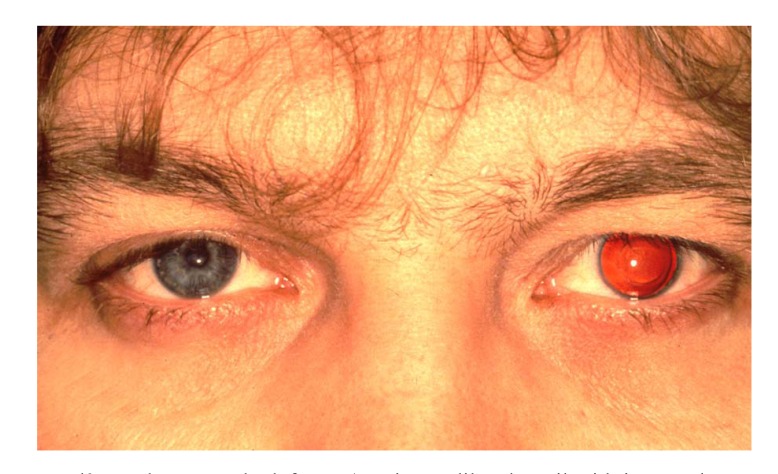
So-called Urrets-Zavalía syndrome on the left eye (persistent dilated pupil with intraocular pressure rise) after Atropine application at the end of keratoplasty for keratoconus without peripheral iridotomy.

A peripheral iridotomy at 12 clock (Fig. **5**) serves as prophylaxis against so-called Urrets-Zavalía syndrome in young people with keratoconus [26]. An iridotomy can avoid a persistently wide pupil, iris atrophy and secondary glaucoma after the administration of atropine with PKP (Fig. **6A**-**C**). We assume that Urrets-Zavalia syndrome results from peri-operative angle closure with a maximum pressure gradient and iris sphincter atrophy resulting from wound leakage.

**Fig. (7):**
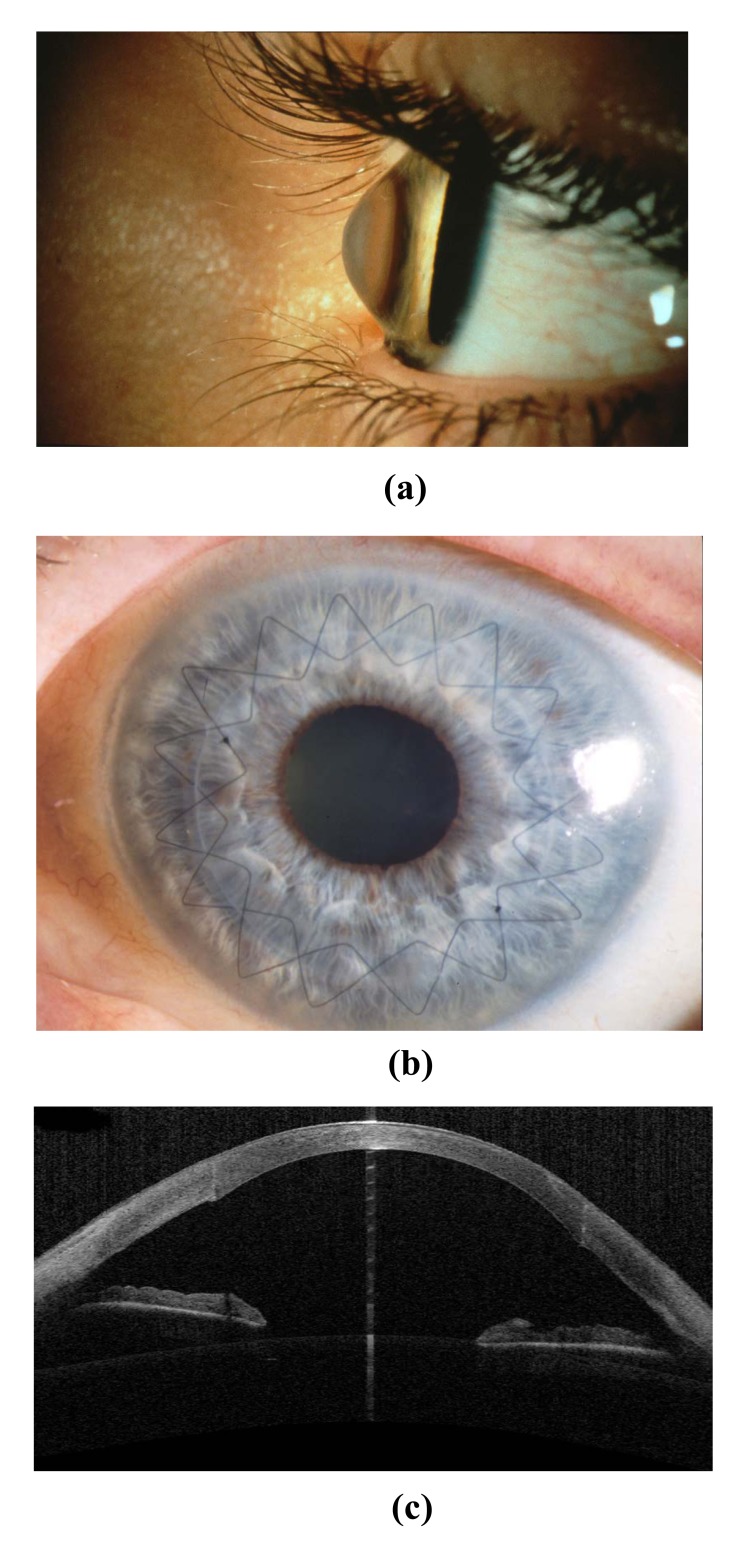
Advanced keratoconus: **(a)** Side view; **(b)** Status post excimer laser keratoplasty (8.0/8.1 mm) with double-running cross-stitch suture according to Hoffmann. **(c)** The anterior segment OCT shows tension-free graft apposition of the perpendicular cut edges without anterior step formation.

**Fig. (8):**
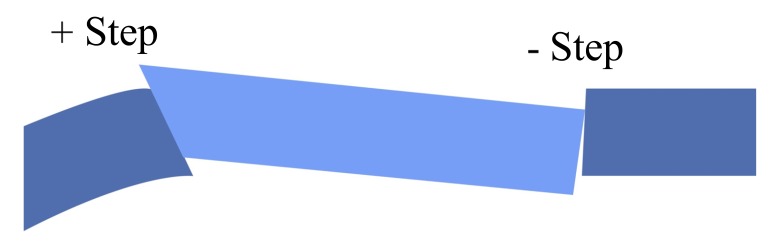
Anterior steps (+ step = donor too high, - step = donor too low) must be avoided during surgery when placing sutures. For peripheral stroma thinning due to underlying disease, posterior steps are often unavoidable in keratoconus (donor overrides host cornea into the anterior chamber).

As long as Bowman's layer is intact, a double running cross-stitch suture according to Hoffmann (Fig. **7A**-**C**) is preferred, since it results in higher topographic regularity, earlier visual rehabilitation and a lower rate of suture loosening [13, 27]. All suture knots are burried in the stroma to avoid mechanical irritation and the induction of neovascularisations. We aim to produce deep stromal “pre-Descemet’s” stitches. In the case of a thinner host cornea, it is inevitable that stitches will penetrate into the anterior chamber. Anterior steps (PLUS or MINUS) in the area of the graft-host-junction must be avoided at all costs (Fig. **8**), posterior steps are allowed, and often unavoidable with keratoconus.

Intra-operative keratoscopy (PLACIDO disk) with tension adaptation of the continuous sutures or re-placement of the simple interrupted sutures should be used after the lid speculum and cardinal sutures have been removed.

### 1.6 Post-Operative Pearls of PKP

In cases of primary graft insufficiency (*i.e.* the graft does not clear up at any time after PKP), the aim should be to replace it at an early stage, *i.e.* after no more than 6 weeks. Here, if the donor tissue has been documented as good at the cornea bank and the surgical technique was uncomplicated, it is always important to consider latent herpes simplex virus infection of the graft as the cause of primary graft failure [28].

So-called “idiopathic endothelial cell loss” after PKP in keratoconus is significantly lower in keratoconus than in Fuchs dystrophy, and again lower than in pseudophakic bullous keratopathy. We attribute this in keratoconus to endothelial migration along a density gradient from the host cornea onto the graft [29].

An immunological graft reaction can occur even after several years [30, 31]. This may be epithelial, stromal or endothelial. Typical of the so-called chronic stromal immune reaction are nummular-like, fine sub-epithelial infiltrates such as in epidemic keratoconjunctivitis. However, in the immune reaction these are restricted to the graft [32]. The stromal immune reaction may occur peracutely in the form of a graft abscess. The most frequent immunological graft reactions are, however, endothelial, either acute diffuse (here the graft becomes completely cloudy) or chronic focal. Here a so-called Khodadoust line forms from an edge of the graft - typically with the presence of neovascularisation - such as a steppes fire over the entire graft to the opposite graft edge. In the case of an immune reaction, one must immediately treat with high-doses of topical prednisolone acetate every half hour. Intracameral Fortecortin injection has proven successful. We prefer an addition of systemic steroids (such as initial 250 mg prednisolone) [13].

Intra-ocular pressure decompensation is not a typical complication after keratoplasty due to keratoconus [33]. Steroid response should be considered in the case of acute ocular hypertension, and steroid application should be modified. In no case is early surgical anti-glaucoma intervention indicated!

### 1.7 Preference for a Non-Contact Trephination Technique

Each mechanical trephination in thinned keratoconus corneas leads to non-round openings in the host cornea due to compression and distortion. Prof. Herbert Kaufman has recommended for more than 20 years not to use an obturator in trephination of the keratoconus with a manual trephine Fig. (**9**). This is not the only reason why trephination today is done preferredly non-mechanically with a laser, especially for keratoconus. In clinical practise, non-mechanical trephination may today be performed with the 193-nm excimer laser (since 1989) or a femtosecond laser (since 2006). In Homburg/Saar and Erlangen, we have, since 1989, successfully trephined more than 1,500 keratoconus eyes with the Zeiss-Meditec MEL60^®^, MEL70^®^ and the Schwind Amaris Excimer laser using metal masks with eight “orientation teeth/notches” [34, 35].

### 1.8 Principle of Excimer Laser Trephination

In donor trepination, a corneoscleral disc (16 mm Ø) is fixed in an artificial anterior chamber and toned to a physiological intraocular pressure. Subsequently, a 0.5 mm thin metal mask is centred and placed in position and a laser beam (Ø approx. 1 mm) is guided by a helium-neon aiming beam moving along the outer edge of the metal mask to the perforation. The central cornea is protected from stray radiation by viscoelastics. In recipient trephination, a 0.5 mm thin recipient mask is placed directly on the patient's cornea. Here the laser beam is moved along the inner edge of the metal mask (half of the laser beam on the metal mask, half of the laser beam on the cornea). Further acceleration and standardisation of excimer laser trephination has been achieved since 11/2014 in Homburg/Saar using the pseudo ring profile of the Schwind-Amaris Excimer laser (750 Hz).

**Fig. (10):**
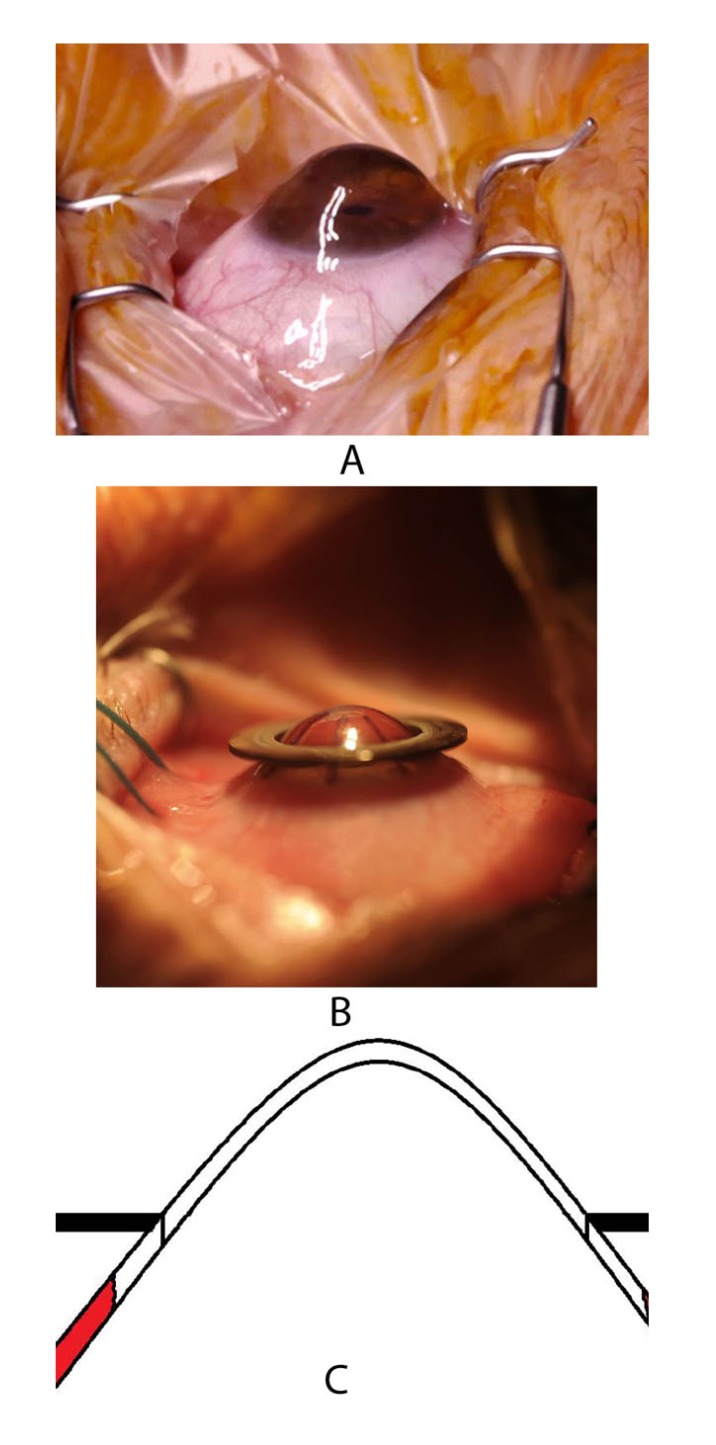
Extremely advanced keratoconus with cone-shaped protruding cornea in the side view (**a**), the recipient mask is freely laid on the corneal cone for excimer laser trepanation (**b**), it rests on the cone like a “ruff” without distorting forces during trephination; schematic presentation (**c**).

A major benefit of excimer laser trephination is the ability to lay the mask freely around the cone-shaped protruding cornea (Fig. **10A**) after having assured the horizontal direction of the limbal plane of the eye. The recipient mask rests on the cone like a “ruff” without exerting distorting forces during trepanation (Figs. **10B**, **C**). Due to the use of the mask technique, an identical configuration of the donor and recipient is assured for both the vertical and horizontal dimensions.

### 1.9 “Orientation Teeth/Notches”

Symmetrical adjustment of the graft in the recipient bed is ensured through so-called orientation teeth/notches [12, 34]. The 8 cardinal sutures are positioned at the orientation teeth/notches, the size of which is 0.3 x 0.2 mm. The major practical advantage of the orientation teeth for the micro-surgeon is the exact positioning of the second cardinal suture to prevent horizontal torsion. In particular, execution of the double-running cross-stitch sutures according to Hoffmann (Fig. **7**) is made considerably easier during the subsequent course of the operation because the first 8 cardinal sutures provide the microsurgeon with a waterproof wound early on.

## 2 RESULTS OF THE PROSPECTIVE RANDOMISED STUDY

After suture removal, the astigmatism in the control group (Geuder Motor Trephine) - at 6.1 ± 2.9 dpt - was twice as high as in the excimer laser group at 3.0 ± 2.1 dpt. The decisive factor is that in the excimer laser group - at 2.8 ± 2.0 dpt - almost the entire keratometric astigmatism had been subjectively tolerated in the spectacles as a cylinder, whereas this was true only for two-thirds in the control group at 4.2 ± 2.4 dpt. This may be dependent upon the regularity of the graft surface. After suture removal, the SRI in the excimer laser group - at 0.91 ± 0.45 - was significantly lower (p< 0.01) compared to the control group at 1.0 ± 0.46. In the excimer laser group, the astigmatism diminished after suture removal by 0.2 ± 3.1 dpt. In the control group it increased by 2.3 ± 3.4 dpt. Overall, there was a decrease in astigmatism in 52% of the eyes of the excimer laser group after suture removal, whereas in the control group there was an increase in astigmatism in 76% of the eyes after suture removal.

Concerning mechanical trephination, the Guided Trephine System GTS with donor and recipient trephination from the epithelial side appears to be the highly beneficial, especially for keratoconus. Objective astigmatism results appear to be only slightly less favourable with GTS than excimer laser trephination [36].

However, the only crucial aspect for the patient is the visual acuity with spectacle correction: After suture removal, the best spectacle-corrected visual acuity in the excimer laser group increased to 0.8 ± 0.2 and was therefore significantly higher than in the control group (0.6 ± 0.2) (P<0.001). These results are of special significance above all for patients with keratoconus, who are usually young, have a career and benefit in particular from early and complete visual rehabilitation [37]. We were able to show in this context that the refractive results after keratoplasty are no less favourable if the operation is only performed upon the occurrence of contact lens intolerance [21].

### 2.1 Trephination with Unstable Cornea and Repeat Keratoplasty

**Fig. (11):**
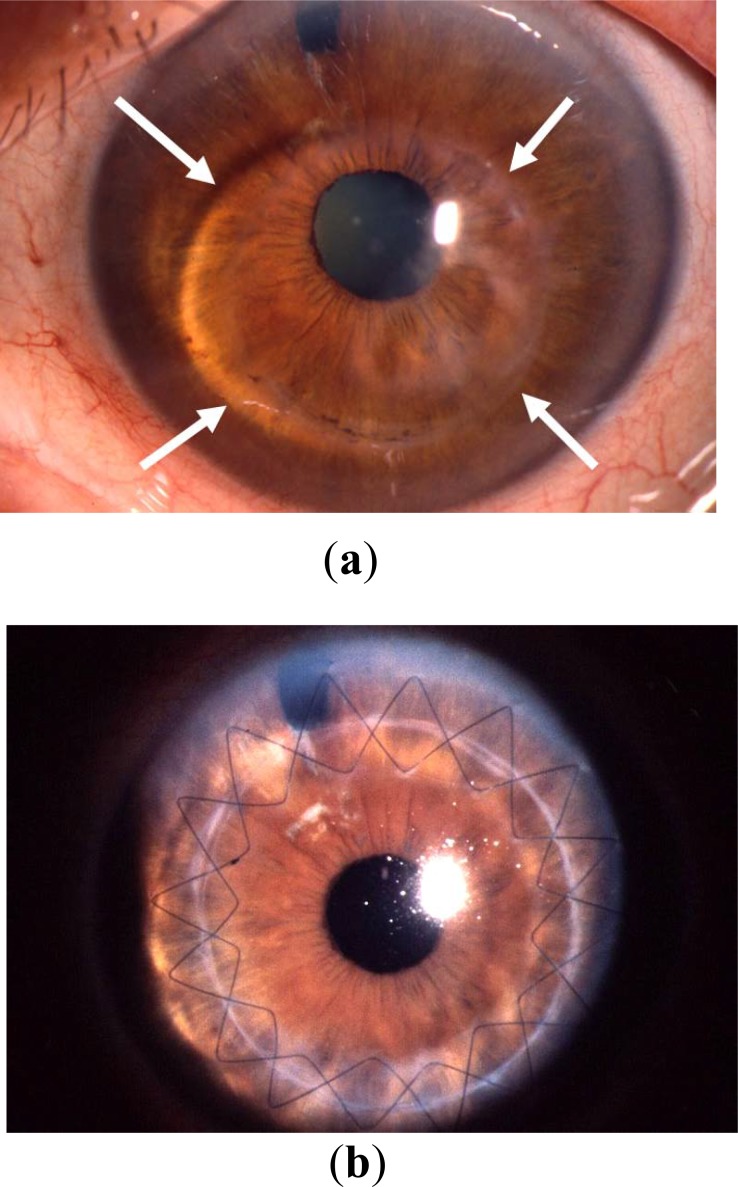
So-called “recurring keratoconus” in too-small, downwards decentered grafts: (a) Before, (b) after repeat PKP with larger, well-centred graft. The high fit precision after 193 nm excimer laser trepanation allows for low suture tension without so-called “barrel-top configuration” at the graft-host-junction and good vision with all-sutures-in.

Last but not least, the 193-nm excimer laser enables non-contact (!) trephination with a primary unstable cornea. This includes, among others, the situation of iatrogenic keratectasia as a consequence of LASIK. With a so-called ‘recurent keratoconus’ where the graft diameter was too small initially, a well-centred repeat keratoplasty with a larger diameter (typically 8.0 or 8.5 mm in the host) allows less suture tension without creating a so-called ‘barrel-top configuration’ at the graft-host-junction with good vision even in the presence of sutures [14] (Fig. **11**).

### 2.2 Femtosecond Laser (FSL) Assisted PKP

The advantages of FSL PKP are that no masks are needed, there is no tissue loss and no thermal effects occur. With real 3-D sections it may be possible to achieve self-sealing wounds. Therapeutic use of FSL has been described in detail by the Freiburg Working Group, among others [38].

### 2.3 Principal Problems with FSL Trephination

**Fig. (12):**
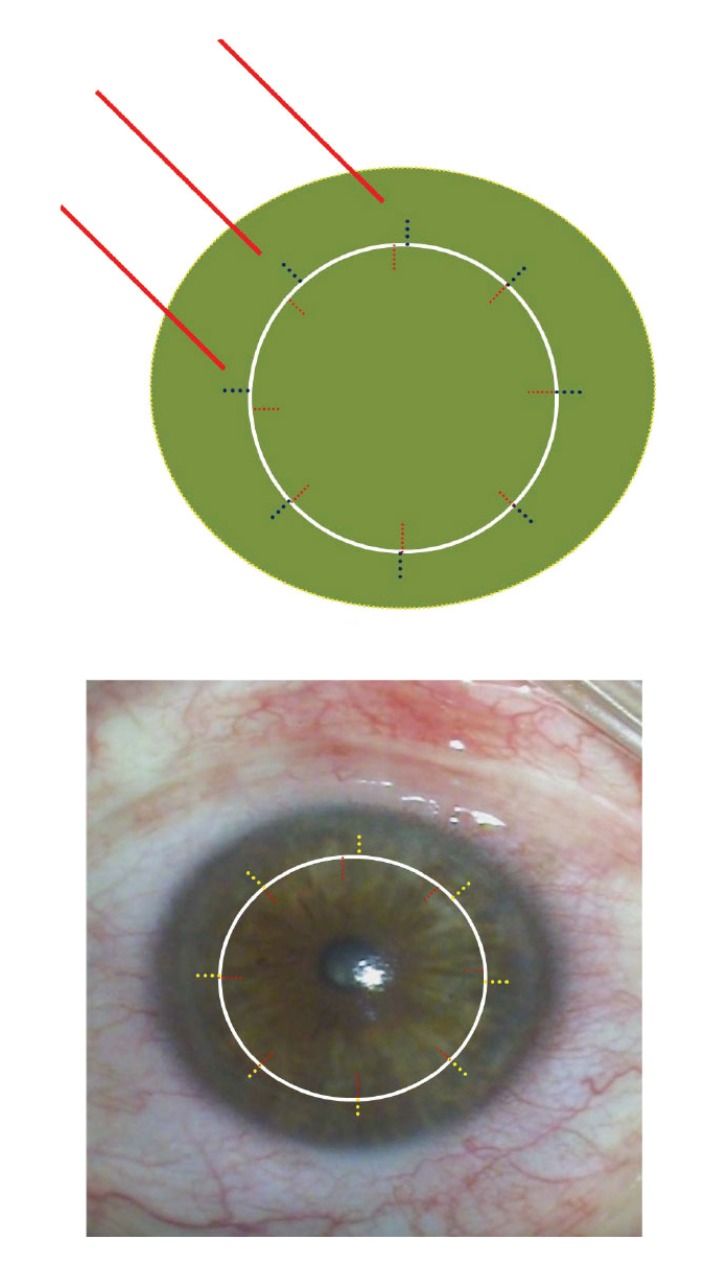
The 8 orientation aids (superficial lines in the donor and recipient in the area of the graft-host-junction) which can, for example, be placed with the Intralase femtosecond laser, are rarely matching completely during femtosecond laser assisted PKP in keratoconus, because a round graft must be adjusted into a non-round host opening after intraoperative applanation of the cone.

**Fig. (13):**
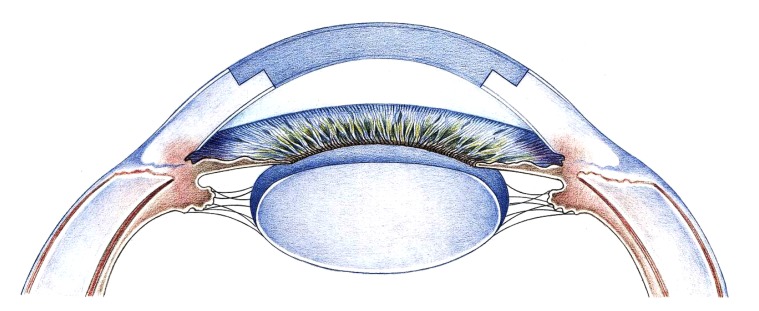
So-called Mushroom configuration in femtosecond laser trephination with keratoconus (courtesy of Prof. Gabriel van Rij, Rotterdam). The larger diameter at the level of the epithelium allows for better corneal curvature, the smaller diameter at the level of the endothelium is intended to help protect the patient’s healthy peripheral endothelium.

Femtosecond laser keratoplasty has caused a good deal of excitement for the past 10 years. The advantages of femtosecond laser keratoplasty are the arbitrary horizontal and vertical shapes, including the “top hat”, “mushroom”, “zigzag”, “Christmas tree”, “octagon”, “decagon”, “dovetail” *etc* [38]. The principal problem with every femtosecond laser trepanation is that local forces are unleashed in the cornea, which cause deformation – especially with a flat, but also with a curved interface. In advanced keratoconus in particular, this results in “non-round” (often oval- or pear-shaped) apertures in the patient's cornea and therefore horizontal torsion as the main intra-operative determinant of high/irregular astigmatism after PKP (see Fig. (**9**), analogue). The eight lines which are applied, for example with the Intralase femtosecond laser, in the donor and recipient can often not be brought into complete alignment intra-operatively in the keratoconus eye [1, 39] (Fig. **12**). There is not much data about the potential advantages of femtosecond laser keratoplasty after suture removal. Only the Freiburg working group has published results pertaining to the situation after complete suture removal. After a mean follow-up of 14±5 ​months, topographic astigmatism without sutures in the mushroom profile was 6.4±3.0 dpt, and in the top hat profile 5.8±4.6 dpt [40]. The amount of astigmatism after femtosecond PKP is, therefore, comparable with that after motor trephination (now withdrawn from the market [36]). In addition, the rate of post-surgical immune reactions is significantly increased with a FSL assisted mushroom profile [31] (Fig. **13**).

### 2.4 Prospective, Randomised EXL vs. FSL Keratoplasty Study

Recruiting for a prospective, randomised study which compares the results between excimer laser- and femtosecond laser-assisted trephination for keratoconus, has been concluded in the Homburg Keratoconus Centre (HKC) (Fig. **2**). The special characteristics of the mushroom technique researched here are shown in the following details: (1) thickness of the anterior peripheral rim is 2/3 of the corneal thickness, (2) donor oversize by 0.1 mm, (3) intended pre-Descemet’s suture placement with a double-continuous cross-stitch suture.

The following main outcome measures were investigated before, and at earliest 2 months after complete suture removal:

- visual acuity with spectacle correction (not contact lens acuity!),
- topographic astigmatism (not only refractive cylinder!),
- topographic regularity

60 patients aged 20 to 81 years (phakic or pseudo-phakic, primary central PKP, one microsurgeon BS) were randomised into four groups in this single-centre study. There were 15 keratoconus and 15 Fuchs-dystrophy patients, each operated with an excimer laser (groups I and II) and with femtosecond laser (groups III and IV). Exclusion criteria included repeat PKP and/or simultaneous lens surgery. A ‘mushroom’ (keratoconus) or ‘top hat’ (Fuchs-dystrophy with stromal scar) profile was created using FSL with an internal diameter of 7.5 mm and an external diameter of 8.5 mm as well as a so-called ‘side cut’ in 2/3 of the corneal thickness. The diameter of the cylindrical profile for the excimer laser PKP was 8.0 mm. The overdimension of the graft was 0.1 mm in all groups.

 Fixation was done in a standardised way with a double,-running cross-stitch suture. With FSL PKP, the stitch depth was intended pre-Descemet’s and not to the plane of the side cut. The first suture were removed after 1 year, the second suture was removed after 1.5 years under topical anaesthesia [39].

The following results were obtained: In particular for keratoconus, the FSL group showed more decentration, more Vis à tergo, and more often individual sutures were required in order to ensure donor-recipient-apposition without steps and gaps [39]. At least 2 months after removal of all all sutures, the topographic astigmatism in keratoconus after FSL trephination (8.1 ± 3.0 dpt) was significantly higher than with EXL trephination (3.2 ± 2.7 dpt). Also, the Surface Regularity Index SRI of the TMS-5 system was significantly more favourable after EXL trephination (0.57 ± 0.40) than after FSL trephination (1.16 ± 0.54). No significant differences between EXC and FSL PKP at the cellular level were found in confocal microscopy [41].

### 2.5 Excimer Laser-Assisted Deep Lamellar Keratoplasty (“Homburg Excimer DALK”)

Deep anterior lamellar keratoplasty (DALK) can be considered as an alternative to PKP in keratoconus. This procedure is surgically demanding and time-consuming. Furthermore, the course of the operation is not anywhere near as possible to standardise as with PKP [42, 43]. It has been established that good visual acuity which is achieved after PKP in keratoconus patients can only be achieved in DALK patients if the patient cornea is freed directly to the Descemet’s membrane. In order to achieve this goal, in our opinion the so-called “Big Bubble Technique” is the most standardised and successful approach [43, 44].

 This was described originally by Anwar [45] in 2002, using an injection of air with a 30 gauge needle directly in front of Descemet’s membrane in the patient’s cornea in order to separate Descemet’s membrane from the corneal stroma. If there is a perforation of Descemet’s membrane, usually one must “convert” to a PKP (incidence: 5-15% depending on the experience of the microsurgeon).

In Homburg/Saar we prepare the donor and recipient trephination with the excimer laser along the metal mask in the typical way [3, 46], but taking into account the Pentacam thickness of the host, we do not perforate the host cornea. The goal is that these typically young keratoconus patients experience no disadvantages if a DALK is planned. If the “big bubble” is successful and we can expose Descemet’s membrane - without perforating and without leaving predescemetal host stroma - we terminate the operation as DALK. If this does not succeed to the operator’s satisfaction, the operation can be completed as classical excimer laser PKP with all of the advantages described above without any disadvantage for the patient [36].

### 2.6 Post-Operative Care After PKP for Keratoconus [11]

We recommend prednisolone acetate AT initially 5 times a day, then taper every 6 weeks by 1 drop to prevent immune reactions. In addition, we recommend nourishing gel 5 times per day (such as Hylogel or Corneregel) because corneal sensitivity is dramatically reduced in the first months after PKP. The first suture is removed after 1 year, the second suture after 1.5 years under outpatient conditions. Additional microsurgical procedures (such as limbus parallel keratotomies and compression sutures, cataract surgery with/without toric IOL, or toric add-on IOLs in pseudophakia patients) or final eyeglass adjustments are carried out at least 6 weeks after complete suture removal.

## CONCLUSIONS FOR CLINICAL PRACTICE

In order to avoid high and/or irregular astigmatism after suture removal, a trephination system should be used for penetrating keratoplasty which ensures symmetrical, tension-free fitting of a circular donor disc in a circular recipient bed with congruent and easily waterproof-adapting incision edges. These demands for optimal trephination are best fulfilled at this time with a non-mechanical excimer laser assisted trephination procedure, which has been conducted for more than 25 years on more than 4,000 patients, and shows proven benefits in regards to topographic astigmatism, regularity of the topography and vision after suture removal. The effort is particularly valuable in (1) young patients with keratoconus – especially after corneal hydrops, (2) repeat keratoplasty (due to high astigmatism), (3) paediatric keratoplasty, (4) unstable cornea (such as after RK, iatrogenic keractasia after LASIK). Femtosecond laser assisted keratoplasty has significantly more expense, and the need for suction and applanation of the conus are combined with intraoperative disadvantages such as decentration and step/gap formation at the graft-host-junction – often requiring additional single sutures at the end of surgery. Superiority of femtosecond laser assisted PKP could not be demonstrated by us and also in the literature over the past 10 years (Table **1**). A few published results in relation to astigmatism and topographic regularity after suture removal demonstrate that the high technical and financial cost of FSL use for PKP - especially in keratoconus - is not justified.

## Figures and Tables

**Fig. (3):**
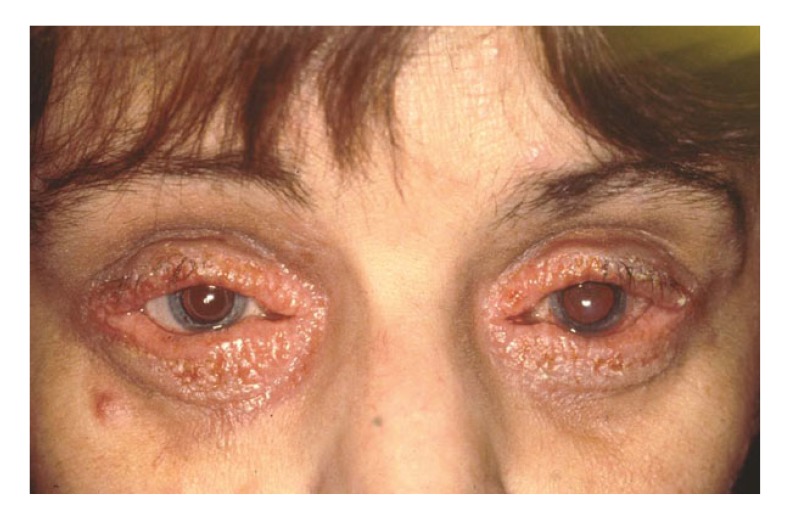
Severe neurodermatitis in a 40-year-old female patient with keratoconus. Consideration should be given as to whether cyclosporin A orally can be administered at a dosage of 150 mg twice a day for 4 weeks before keratoplasty. Prior to surgery, conventional eyelid margin hygiene and a dermatological consultation are obligatory.

**Fig. (9):**
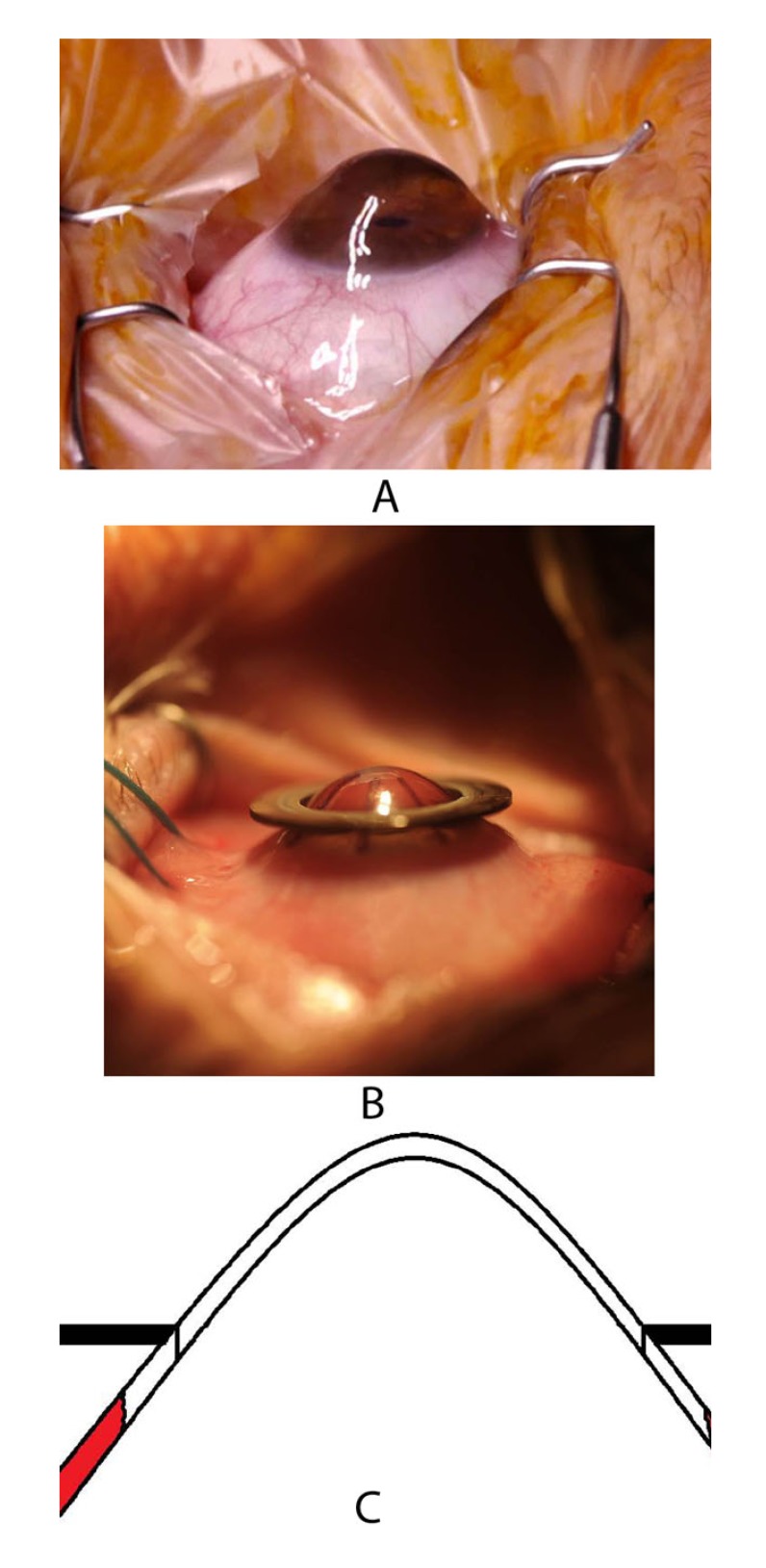
As recommended by Prof. Dr. Herbert Kaufman over more than 20 years, one must not use an obturator during trephination of keratoconus with a hand-held trephine, because this leads to oval or pear-shaped incisions in the host cornea due to applanation during trephination.

**Table 1:** Benefits and practical aspects of excimer laser (EXL) and femtosecond laser (FSL) for keratoplasty with keratoconus (+++ = very beneficial, - - - = very unfavourable).

	EXL	FSL
“Cumbersome procedure”	+	- -
Centration	+ + +	+
Avoidance of deformation and compression	+ + +	- - -
Higher IOD during the laser action	+ + +	-
Minimisation of cut completion with scissors	(+)	+ +
Undisputed localisation of the first 8 cardinal sutures	+ + +	+
Stable anterior chamber during suturing	++	+ + +
Feasibility to use a double continuous suture	+ + +	+ + +
Additional single sutures not necessary	+ + +	+
Possible trephination with an instable cornea	+ + +	- - -
Possible trephination with repeat keratoplasty	+ + +	-
Helpful for DALK	+ +	+ +
Immune reactions	+	--
